# Disagreement between splenic switch-off and myocardial T1-mapping after caffeine intake

**DOI:** 10.1007/s10554-017-1274-0

**Published:** 2017-11-24

**Authors:** Dirkjan Kuijpers, Randy van Dijk, Marly van Assen, Theodorus A. M. Kaandorp, Paul R. M. van Dijkman, Rozemarijn Vliegenthart, Pim van der Harst, Matthijs Oudkerk

**Affiliations:** 1 Center for Medical Imaging, University of Groningen, University Medical Center Groningen, Hanzeplein 1 EB 45, Groningen, The Netherlands; 2 Department of Cardiology, University of Groningen, University Medical Center Groningen, Groningen, The Netherlands; 3 Department of Cardiovascular Imaging HMC-Bronovo, The Hague, The Netherlands; 4 Department of Radiology, University of Groningen, University Medical Center Groningen, Groningen, The Netherlands

**Keywords:** T1-mapping, Stress CMR, Splenic switch-off, Adenosine, Cardiac magnetic resonance

## Abstract

Caffeine is an adenosine receptor antagonist and a possible cause of inadequate stress perfusion. Splenic switch-off (SSO) and splenic rest-stress T1-mapping have been proposed as indicators of stress adequacy during perfusion cardiac magnetic resonance (CMR). We compared myocardial rest-stress T1-mapping with SSO and splenic rest-stress T1-mapping in patients with and without recent coffee intake. We analyzed 344 consecutive patients suspected of myocardial ischemia with adenosine perfusion CMR. All 146 normal CMR studies with a normal T1-rest of the myocardium, used as standard of reference, were included and divided in two groups. 22 patients accidentally ingested coffee < 4 h before CMR, compared to control group of 124 patients without self-reported coffee intake. Two independent readers graded SSO visually. T1-reactivity (ΔT1) was defined as percentual difference in T1-rest and T1-stress. Follow-up data were extracted from electronic patients records. In patients with recent coffee intake SSO was identified in 96%, which showed no significant difference with SSO in controls (94%, p = 0.835), however event rates were significantly different (13.6 and 0.8%, respectively (p < 0.001), median FU 17 months). Myocardial ΔT1 in the coffee group (− 5.2%) was significantly lower compared to control (+ 4.0%, p < 0.001), in contrast to the splenic ΔT1 (− 3.7 and − 4.0%, p = 0.789). The splenic T1-mapping results failed to predict false negative results. SSO and splenic rest-stress T1-mapping are not reliable indicators of stress adequacy in patients with recent coffee intake. Therefore, the dark spleen sign does not indicate adequate myocardial stress in patients with recent caffeine intake. Myocardial rest-stress T1-mapping is an excellent indicator of stress adequacy during adenosine perfusion CMR.

## Introduction

Myocardial adenosine perfusion with cardiovascular magnetic resonance (CMR) imaging is state of the art in the diagnosis of myocardial ischemia [1]. Stress perfusion CMR is the most sensitive non-invasive imaging test for detecting myocardial ischemia and provides important diagnostic and prognostic information [2–4]. Adequate myocardial stress is essential for the detection of myocardial ischemia, with adenosine being the most widely used vasodilator agent. Current guidelines indicate that caffeine containing substances, such as coffee, are nonselective adenosine receptor antagonists that may cause false negative perfusion CMR results [1]. Conventional hemodynamic markers of myocardial stress response, such as heart rate and systolic blood pressure correlate poorly with changes in myocardial perfusion and are unreliable predictors of stress adequacy [5].

Native (non-contrast) rest-stress T1-mapping of the myocardium has proven to be a reliable biomarker of adenosine stress [6–8]. Native T1-mapping allows quantification of myocardial water content and is closely related to myocardial blood volume [6–8]. The change of myocardial blood volume during perfusion CMR can be expressed as a percentile increase or decrease of T1 from rest to stress (T1-reactivity, ΔT1). A negative myocardial ΔT1 has shown to be a predictor for recent coffee intake before adenosine perfusion CMR [7, 8].

The visual splenic switch-off (SSO) is another predictor for stress adequacy. It has been reported that failed splenic switch-off (SSO) during adenosine stress perfusion CMR occurred more frequently in patients with proven false negative CMR results as compared to patients with true negative results [9]. The same principle of native T1-mapping during rest and stress can also be applied to the spleen. Splenic T1-mapping during administration of adenosine was recently introduced to predict the occurrence of SSO [10].

In this study we compared myocardial rest-stress T1-mapping as a marker of adenosine stress response with SSO and rest-stress T1-mapping of the spleen in patients with a normal stress CMR study with and without recent coffee intake. Secondly, we assessed the value of SSO as stress biomarker for major adverse cardiovascular events (MACE). We hypothesized that in contrast to SSO and rest-stress T1-mapping of the spleen, rest-stress T1-mapping of the myocardium enables the identification of patients with insufficient adenosine stress due to recent coffee intake.

## Methods

### Study population

In this study we identified 344 consecutive patients suspected of myocardial ischemia, with adenosine stress perfusion CMR between August 2015 and April 2016. The institutional review board approved this study. An overview of the patient inclusion flow chart is shown in Table 1. Of these 344 patients, 20 eligible patients did not give informed consent and were not included. There is some overlap of the study population with previously published papers by our group because this study was part of the multiparametric MR trial [7, 8]. Rest-stress T1-mapping of the myocardium as a marker of adenosine stress response, was compared with SSO and rest-stress T1-mapping of the spleen for the assessment of myocardial stress adequacy during adenosine perfusion CMR. To avoid T1-mapping measurements errors and interference with other (cardiac) diseases, all abnormal CMR studies were excluded and a normal native T1-rest value (± 2SD) of the myocardium (977 ± 80 ms) was used as standard of reference for the study population [7]. In this cohort 178 of 324 patients were excluded, because of abnormal stress perfusion CMR results, technical failures or other reasons. All anti-angina medication was stopped 4 days before the stress perfusion examination and all patients avoided potential adenosine agonists for at least 24 h. Dipyridamole had to be stopped and, if not possible, was considered a contraindication. As part of the stress perfusion CMR procedure patients were specifically questioned on the use of coffee, consumed less than 4 h before the CMR study.

**Table 1:**
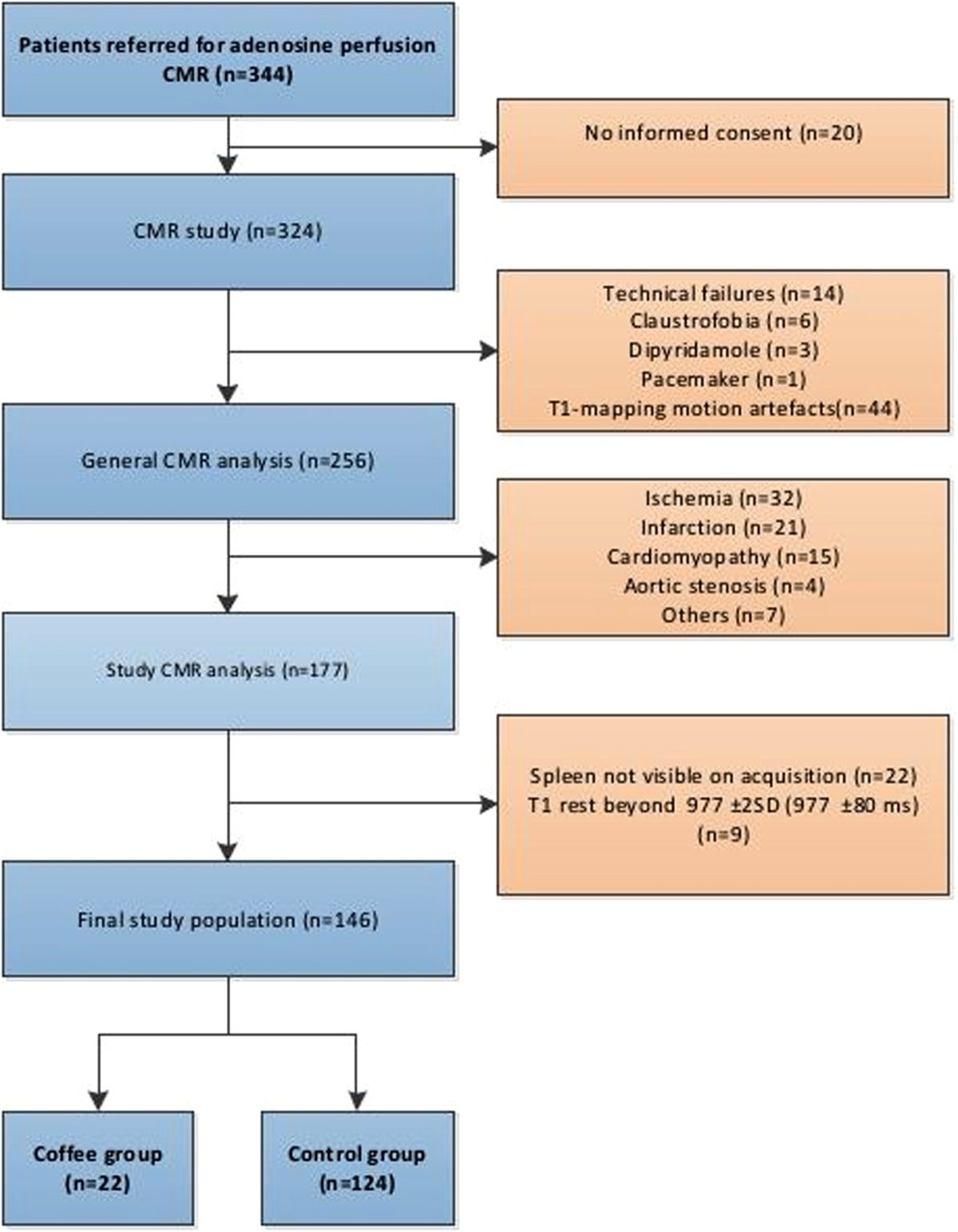
Flowchart of the study population

The remaining 146 patients with normal stress perfusion CMR results (scan reported as normal with no signs of myocardial ischemia, no Gadolinium enhancement and normal T1-mapping of the myocardium), were divided in two groups, with (n = 22) and without (n = 124) recent coffee intake (one or two cups of coffee).

The two groups were followed for MACE, acquired from the electronic patient records. MACE was defined as myocardial ischemia, myocardial infarction, heart failure or cardiac death. Results of 15 of the patients from the coffee group and 50 controls in this study population were previously reported [7].

### CMR protocol

Adenosine perfusion CMR was performed on a 1.5T system (Magnetom Avanto-fit; Siemens Healthineers, Erlangen, Germany). Native T1-mapping was performed using an investigational Modified Look-Locker Inversion Recovery (MOLLI) based T1–mapping sequence (WIP780B) at rest and during adenosine stress. A 5(3)3 sampling scheme was performed, including eight images in 11 heartbeats. For the MOLLI acquisition an initial inversion time of 110 ms was used with an 80 ms increment. A pixel-wise T1 map of the myocardium was acquired with inline motion correction. The images were generated using a single-shot steady-state free-precession readout. Typical parameters were: field of view, 300 × 256 mm^2^; TR 279 ms; TE 1.1 ms; slice thickness 8 mm; acquisition matrix, 192 × 128; in plane spatial resolution, 1.4 × 1.4 mm^2^; bandwidth, 1085 Hz/pixel; flip angle, 35° and parallel imaging acceleration factor of 2. T1 maps of the myocardium (mid-ventricular) and spleen were acquired at rest and during peak dose adenosine stress. Directly after acquiring the T1-maps, a nonselective saturation recovery perfusion sequence was started during the first pass of 0.1 mmol/kg macrocyclic Gadolinium injected at a flow rate of 5 ml/s and flushed with 25 ml 0.9% NaCL, after 3 min of adenosine infusion (0.140 mg/kg/min). Perfusion series were analyzed as previously described [11]. The perfusion images were followed by conventional late enhancement images (LGE).

### Visual assessment of splenic switch-off

The visual analysis includes a simple visual comparison of the enhancement of the spleen during the perfusion at stress. The spleen was viewed in one or more stress perfusion short-axis sections in which the spleen was seen best. The enhancement of the spleen was analyzed at the start of the perfusion, before the arrival of the contrast agent (baseline) and 10–40 s after the administration of the contrast agent. Perfusion was graded as either *splenic switch-off* (ie, clearly visually no splenic enhancement compared to baseline) or *no splenic switch-off* (ie, clearly visually splenic enhancement compared to baseline). The images were assessed independently by two radiologists (TK, DK) both with more than 10 years of experience in CMR imaging. Observers were blinded to the information on coffee intake and T1-mapping results. Disagreements (in four patients) between the observers were settled in consensus.

### Rest-stress T1-mapping analysis

The T1-maps, generated on the imaging console, were analyzed on commercially available software (MASS analytical software, Medis Suite 2.1, Medis, Leiden, The Netherlands). For the myocardial rest-stress T1-mapping assessment, short-axis T1-maps were manually contoured using conservative septal sampling [12]. To avoid partial volume effects of the blood pool, all samples were located in the core of the region of interest (ROI). Regions of myocardial infarction were avoided. T1-rest mapping measurements of the myocardium, used as standard of reference, were performed in multiple segments of the left ventricle. For the splenic T1-maps an ROI was placed clearly inside the spleen with consideration of potential causes of partial volume effects. The measurements of the T1 relaxation times were performed with MASS mapping software, blinded to the patient data. T1-reactivity (ΔT1) was expressed as percentages. T1-rest and T1-stress represent mean T1 values at rest and during adenosine stress, respectively.

### Statistical analysis

Continuous variables without normal distribution were compared using the Mann–Whitney U test in case of unpaired data and the Wilcoxon signed rank test in case of paired data. Unpaired categorical variables were compared using the Chi-squared test. The survival free of MACE was calculated by the Kaplan–Meier method and compared between groups using log rank test. Cox regression analysis was performed to assess possible influence of covariates on event free survival. Inter-observer agreement for the assessment of SSO was assessed by calculating Cohens’ kappa. Statistical significance was defined as a p-value of < 0.05. Statistical analyses were performed using SPSS statistics 23 (IBM corporation, USA).

## Results

**Fig. 1:**
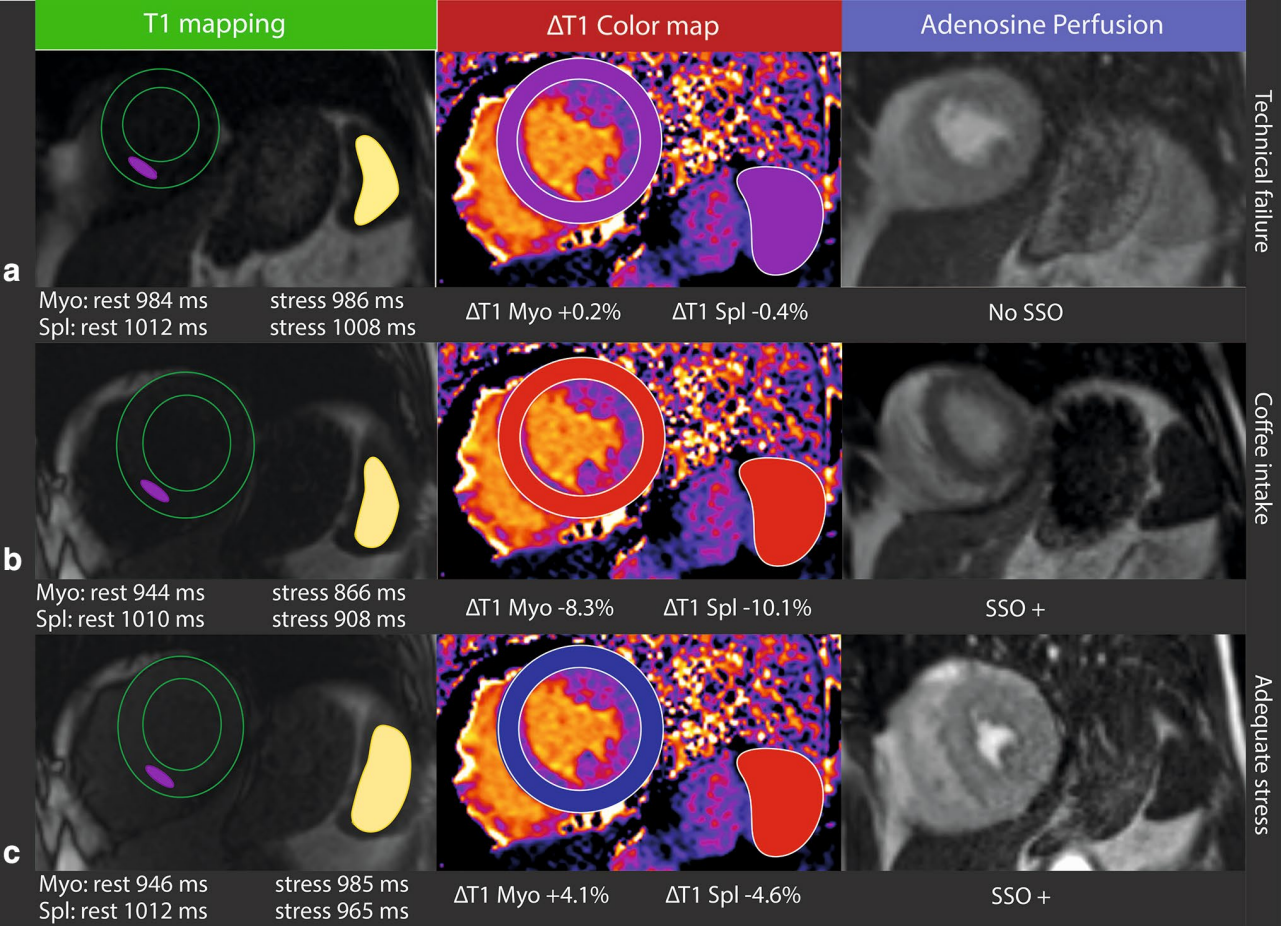
Illustrative presentation of T1-mapping results and SSO assessment based on a case of a 75-year old female that underwent multiple adenosine perfusion CMR evaluations within a short period of time due to: **a** technical failure during the first examination with leakage of adenosine through the extension set of the intravenous line causing inadequate adenosine induced hyperemia, **b** a second examination (14 days later) in which ingestion of coffee < 4 h examination was retrospectively determined and **c** a third final examination 7 days after the second examination without protocol conflicts. For illustrative purposes we present (from left to right) myocardial and splenic native T1-mapping analysis with endo- and epicardial contours graphically visualized (green lines) and regions of interest placed in the inferior septal region of the myocardium (pink) and in the spleen (yellow) with the use of dedicated software, a graphical illustration of the ΔT1 color maps and adenosine perfusion images. **a** Inadequate myocardial stress as shown by a very low ΔT1 of the myocardium (+ 0.2%, purple), minor stress changes of the spleen (purple) and no SSO. **b** Inadequate myocardial stress due to coffee intake in the hours prior to the examination as indicated by the inverted ΔT1 of the myocardium (− 8.3%, red). Strongly negative ΔT1 in the spleen (red) and presence of SSO. **c** Adequate myocardial stress with a positive ΔT1 of the myocardium (blue), adequate stress changes of the spleen (red) and SSO

Characteristics and hemodynamics of the patients are summarized in Table 2. The spleen was visible and feasible for T1-mapping in 88% of the remaining 177 studies, used for study CMR analysis (Table 1). An illustrative presentation of T1-mapping results, ΔT1 and SSO assessment in a case of a 75-year old female that underwent multiple adenosine perfusion CMR evaluations is shown in Fig. 1.

**Table 2:** Patient baseline characteristics and hemodynamics during adenosine stress perfusion CMR

	Controls	Coffee < 4H^a^	p value
	N = 124	N = 22	
Male, n (%)	63 (51%)	13 (59%)	
Age, years	65 ± 11	65 ± 10	0.730
Body weight, kg	80 [67;88]	80 [71;96]	0.377
Resting heart rate, beats/min	74 [67;83]	67 [65;76]	0.050
Stress heart rate, beats/min	88 [77;100]	84 [78;89]	0.104
Rest systolic blood pressure, mm Hg	143 [127;156]	141 [121;159]	0.590
Rest diastolic blood pressure, mm Hg	82 [76;89]	81 [75;88]	0.747
Stress systolic blood pressure, mm Hg	135 [123;152]	135 [125;146]	0.844
Stress diastolic blood pressure, mm Hg	80 [73;85]	78 [72;83]	0.333

^a^Results of 15 of the patients from the coffee group and 50 controls in this study population were previously reported [7]

### SSO and T1-mapping of the spleen

SSO was identified in 95.5% in patients with recent coffee intake and in 94.4% of the control group, without significant difference (p = 0.835) (Table 3). Inter-observer agreement for assessment of SSO was substantial (κ: 0.736).

**Table 3:** T1-mapping values. T1 values were measured at rest (T1 rest) and stress (T1 stress) and T1-reactivity (ΔT1) was calculated [(T1 stress/T1 rest) × 100%] for ROIs in both the septal myocardium and splenic tissue. Visual splenic switch-off was graded by two independent observers

	Controls^a^	Coffee intake < 4 H^a^	p-value
	N = 124	N = 22	
T1_myocard_ rest, ms	977 [956;994]	963 [955;991]	0.457
T1_myocard_ stress, ms	1015 [998;1045]	924 [879;946]	< 0.001
Myocardial ΔT1, %	4.0 [2.8;5.7]	− 5.2 [− 8.1;− 3.6]	< 0.001
T1_spleen_ rest, ms	1022 [994;1078]	1028 [994;1067]	0.943
T1_spleen_ stress, ms	987 [960;1031]	993 [941;1021]	0.520
Splenic ΔT1, %	− 4.0 [− 5.3;− 2.5]	− 3.7 [− 6.7;− 2.5]	0.789
Splenic ΔT1, ms	− 40 [− 59;− 25]	− 38 [− 71;− 26]	0.810
Splenic ΔT1 ≥ 30 ms, n (%)	87 (70)	15 (68)	0.852
SSO, n (%)	117 (94)	21 (96)	0.835

Splenic ΔT1 in ms and ΔT1 ≥ 30 ms are also reported

^a^Myocardial T1-mapping results of 15 of the patients from the coffee group and 50 controls in this study population were previously reported [7]

Splenic T1 values during rest and stress and splenic ΔT1 (%) did not differ significantly when comparing patients with recent coffee intake (1028 [994;1067] ms, 993 [941;1021] ms and − 3.7 [− 6.7;− 2.5]%) with patients in the control group (1022 [994;1078] ms, 987 [960;1031] ms and − 4.0 [− 5.3;− 2.5]% at p = 0.943, 0.520 and 0.789, respectively).

There was no significant difference in both splenic ΔT1 (ms) and the threshold of ΔT1 ≥ 30 ms when comparing patients with recent coffee intake to patients in the control group (p-value: 0.810 and 0.852, respectively). Sensitivity and specificity of the ΔT1 of ≥ 30 ms threshold for predicting SSO in our study population were 74% (66–81%) and 100% (63–100%).

### Myocardial T1-mapping

**Fig. 2:**
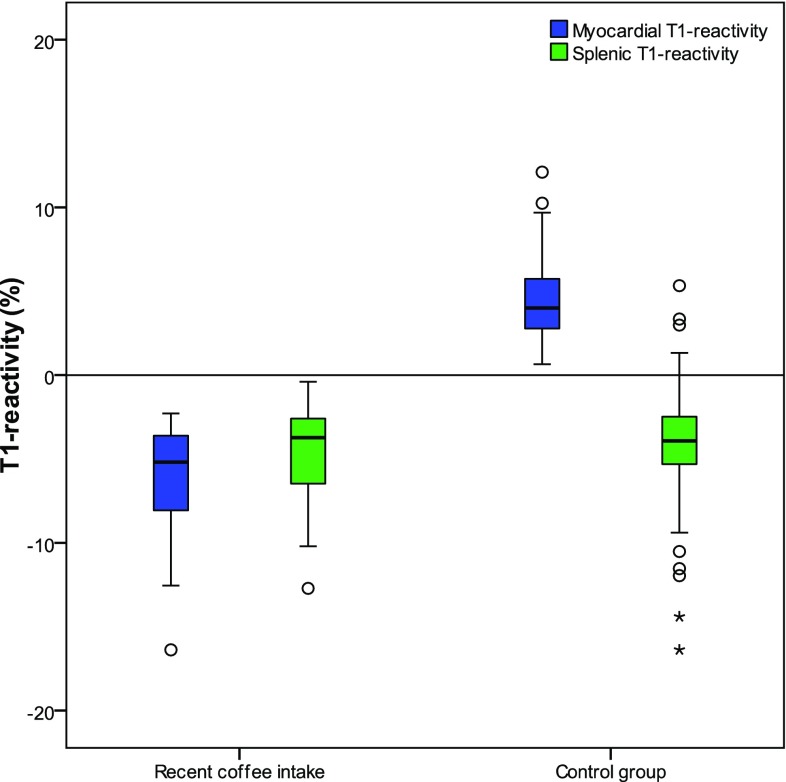
Boxplot of myocardial and splenic T1-reactivity (ΔT1) in patients with coffee intake < 4 h prior to the examination and patients with normal perfusion CMR and no recent coffee intake. There was a significant difference between myocardial and splenic ΔT1 in patients with normal perfusion CMR. No statistical difference was present in patients with coffee intake < 4 h prior to the examination

Myocardial T1 values in rest showed no significant difference in patients with recent coffee intake when compared to the control group (963 [955;991] ms vs. 977 [956;994] ms at p-value: 0.457). Myocardial T1 values during stress were significantly lower in patients with recent coffee intake as compared to the control group (924 [879;946] ms vs. 1015 [998;1045] ms at p-value: <0.001). The myocardial ΔT1 was also significantly lower in patients with recent coffee intake as compared to control (− 5.2 [− 8.1;− 3.6]% vs. 4.0 [2.8;5.7]% at p-value < 0.001). A boxplot of both myocardial and splenic ΔT1 is shown in Fig. 2.

### Follow-up results

**Fig. 3:**
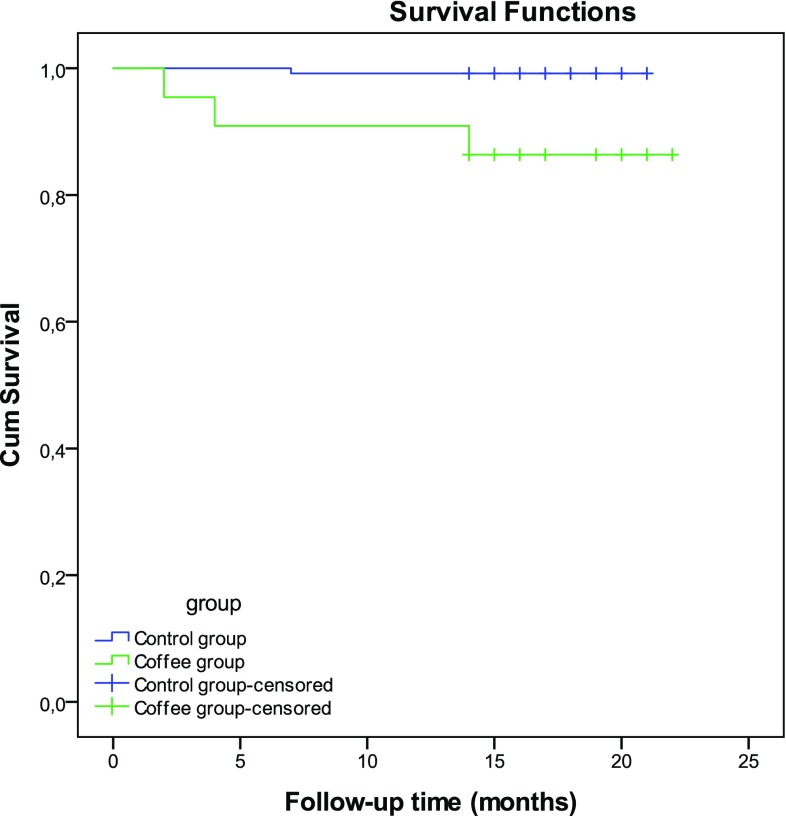
Kaplan–Meier survival curve comparing patients with recent coffee intake to patients in the control group. MACE was defined as myocardial ischemia, myocardial infarction, heart failure or cardiac death. Log-rank test p-value < 0.001

The median follow-up period was 17 months for both groups. In the follow-up period four events were identified. The control group (n = 124) showed one event (0.8%) and the group of patients with recent coffee intake (n = 22) three events (13.6%) with an annual MACE rate of 9.1%. The log rank test showed significant difference in survival from MACE between the two groups (p < 0.001) and cox regression analysis showed no significant influence of age and gender on survival. The Kaplan–Meier curve is presented in Fig. 3.

Two of the three events in the coffee group occurred within 4 months after the initial CMR examination. The first patient (female, 74 year) had persistent atypical angina after the initial perfusion CMR study. The adenosine stress study was repeated 2 months after the first study (after 2 days without coffee) and showed transmural ischemia (four segments) in the anterior wall. The ΔT1 of the myocardium recovered from − 5.4% on the initial scan to + 2.4% on the repeated CMR scan. SSO was present on both studies and the ΔT1 of the spleen was − 6.4 and − 3.6% respectively. Invasive Coronary Angiography (CAG) confirmed a significant stenosis in the left anterior descending artery (LAD). The second and third patient showed persistent atypical angina and were referred for CAG, which showed significant coronary artery disease (CAD). All patients were treated by a PCI procedure. In retrospect we could not detect these ischemic perfusion findings on the initial stress studies.

In the control group one event occurred 7 months after the initial CMR study (with SSO), probably due to progressive arteriosclerotic disease. This patient (female, 77 year) with new complaints of angina pectoris, showed significant single vessel CAD disease with invasive CAG and was treated with a PCI procedure.

## Discussion

### Splenic switch-off

In this study, rest-stress T1-mapping of the myocardium as a biomarker of stress adequacy was compared to SSO and rest-stress T1-mapping of the spleen, both proposed as CMR markers of adenosine stress. We demonstrated that absence of enhancement of the spleen (SSO, spleen appears black), was found in 94% of the patients in the control group, but also in 96% of the patients with recent coffee intake, in which we initially expected enhancement of the spleen (spleen appears bright), due to the antagonistic properties of caffeine on adenosine receptors. Caffeine is a central nervous system stimulant, but is also increases sympathetic activity which may lead to direct vasoconstriction of both myocardial and splenic capillary tissue, as is shown by the negative stress T1-mapping results of both organs in this study [13].

It is reported, that SSO should be present in about 90% of the adenosine perfusion CMR studies with true negative results [9]. In this study the amount of induced adenosine stress in the control group of patients with a true negative outcome, was sufficient to acquire adequate splenic vasoconstriction, represented by a high percentage of SSO (94%).

Absence of SSO was reported to occur four times more often in patients with false negative perfusion CMR scans when compared to invasive CAG in detecting myocardial ischemia [9]. However, even a present SSO is not a guarantee for an optimal stress perfusion CMR result. Our study shows that SSO is not reliable to identify those patients with recent coffee intake, which may lead to false negative interpretation of adenosine perfusion CMR examinations instead of identifying insufficient stress perfusion.

Because the spleen is thought to be visible on most of the short axis perfusion CMR images it may provide a simpler visual assessment of stress adequacy. Although we used a standard CMR perfusion technique, with vendor guided acquisition planes, the spleen was only visible in 88% of the CMR studies in this study. This is lower compared to a former study where the spleen was visualized in 99% of CMR studies [9].

### Rest-stress T1-mapping of the spleen

To optimize the adenosine stress protocol splenic rest-stress T1-mapping was recently introduced to predict the occurrence of SSO during the administration of adenosine [10]. This stress T1-mapping value provides a tool to prolong adenosine administration before the perfusion sequence to increase the stress induced hyperemia and to increase diagnostic accuracy. A decrease of ≥ 30 ms between T1-rest and T1-stress (ΔT1) was found to be the optimal threshold to predict occurrence of SSO during Gadolinium-mediated stress perfusion imaging [10]. In our study, the previously reported optimal cut-off for splenic ΔT1 to predict the occurrence of the SSO (ΔT1 ≥ 30 ms) showed high specificity, but only moderate sensitivity, to predict the SSO. Furthermore, rest T1-mapping, stress T1-mapping and splenic ΔT1 also showed no significant difference between patients with and without recent coffee consumption.

### Patient outcome

In the control group of patients without recent coffee intake, stress adequacy was well predicted by the positive ΔT1-mapping results of the myocardium and in agreement with SSO and the negative ΔT1-mapping results of the spleen. The event rate in this group was very low and comparable with other studies [11, 14].

In the patients with recent coffee intake we found in this adenosine perfusion CMR study an annual MACE rate of 9.1%. Although the median follow-up in the coffee group was 17 months, two of three events in the coffee group occurred already within 4 months after the negative reported stress CMR study. The short period of time between the event and the initial CMR study and the fact that we could not detect any perfusion defect retrospectively, favors caffeine as the primary cause for the false negative results. In contrast to the ΔT1 of the myocardium, both splenic stress markers were not able to identify inadequately stressed patients who are at risk of false-negative findings on adenosine perfusion CMR scans in this study. Therefore, the dark spleen sign does not indicate adequate myocardial stress in patients with recent caffeine intake.

### Limitations

The main limitation of this study is the small study population because the prevalence of patients with coffee intake in the hours preceding the scan was low. Due to the small study population the prevalence of false negative CMR perfusion results (in both the < 4 h coffee group as well as the control group) was also low. The results of the survival analysis should therefore be interpreted with caution. However, they do seem to indicate a difference between the groups when looking at event free survival. Patients were asked to refrain from caffeine for at least 24 h before the CMR appointment, however other (energy) drinks can contain significant amounts of caffeine, which could interfere with our results. The most important limitation of the used MOLLI [5(3)3] technique are motion artefacts. Acquisitions with mapping motion artefacts were excluded from the analysis (13.5% of CMR studies). The exclusion of these acquisitions might be a potential source of selection bias. To validate our results a prospective randomized controlled trial in a larger patient population would be necessary preferably with fixed doses of caffeine to assess the diagnostic accuracy of the various indicators of stress adequacy during adenosine perfusion CMR.

### Conclusion

This study indicates that it is important to ask patients, referred for adenosine stress perfusion CMR, specifically for the intake of coffee less than 4 h before the study, to avoid inadequate stress and to decrease the risk of false negative findings. SSO and splenic rest-stress T1-mapping are unreliable indicators of stress adequacy in patients with recent coffee intake. Myocardial rest-stress T1-mapping is an excellent indicator of stress adequacy during adenosine perfusion CMR.
